# Polygenic risk score in cirrhosis: Does the etiology matter?

**DOI:** 10.1097/HC9.0000000000000541

**Published:** 2025-01-16

**Authors:** Marta Alonso-Peña, Maria Teresa Arias Loste, Joaquin Cabezas, Paula Iruzubieta, Javier Crespo

**Affiliations:** Gastroenterology and Hepatology Department, Clinical and Translational Research in Digestive Diseases, Valdecilla Research Institute (IDIVAL), Marqués de Valdecilla University Hospital, Santander, Cantabria, Spain

To the editor,

We have read with interest the recently published work by Schwantes-An et al^1^ in your esteemed journal. These authors developed a polygenic risk score (PRS) to predict the risk of developing alcohol-associated liver cirrhosis (ALC) among heavy drinkers of European ancestry. A 20 single-nucleotide polymorphism PRS (PRS_ALC_) effectively stratified the risk of ALC. PRS_ALC_ improved ALC risk prediction when combined with clinical predictors and showed potential for predicting metabolic dysfunction–associated steatotic liver disease (MASLD) cirrhosis. Moreover, 3 single-nucleotide polymorphisms (affecting *PNPLA3*, *HSD17B13*, and *TM6SF2*) stand out in PRS_ALC_, which have already demonstrated a strong association with the progression of MASLD. Therefore, the shared genetic background between alcohol-associated liver disease (ALD) and MASLD is emphasized.

We believe that the clinical potential of PRS_ALC_ extends beyond ALD, as the gene variants included in PRS_ALC_ may contribute to hepatic fibrogenesis independently of their effect on hepatic fat.^2^ However, an accurate evaluation of alcohol consumption, not only in patients with ALD, is essential to establish the risk of developing cirrhosis. One limitation of the study was the lack of an objective assessment of alcohol consumption. This could explain the PRS’s ability to stratify the risk for MASLD cirrhosis, as these patients could be unidentified or poorly characterized heavy drinkers. This is critical because the collection of alcohol consumption data is complex and often underreported in some cohorts, such as the UK Biobank. Furthermore, increasing evidence suggests that even small amounts of alcohol may contribute to the progression of MASLD.^3^ We had the opportunity to analyze the impact of different levels of alcohol intake in patients with steatotic liver disease. We observed that low alcohol consumption is very common in patients with MASLD and, especially in the presence of different comorbidities, significantly increased the risk of advanced liver disease.^4^

MASLD has a strong hereditary component, and the genetic architecture of this disease is still not definitive. Recognizing the contribution from these *loci* enhances our understanding, which could primarily apply to some forms of MASLD but probably not all. The new steatotic liver disease nomenclature and diagnostic criteria have established a new category, metabolic and alcohol-associated liver disease, for individuals with MASLD who consume larger amounts of alcohol. It is highly probable that the confluence of alcohol and metabolic disorders could also justify the prognostic capacity of PRS_ALC_ in this newly defined population, highlighting a previously underreported population group. Therefore, it would be interesting to evaluate the prognostic value of PRS_ALC_ in patients with metabolic and alcohol-associated liver disease.

To confirm these aspects, we should strive to improve the accurate quantification of our patients’ alcohol consumption, probably using biomarkers that are already available.^5^ In addition, this study should be extended to other populations, including non-European populations and those with cirrhosis of other etiologies (viral, autoimmune, or cholestatic).

We extend our sincere congratulations for the insightful research. Identifying these new risk *loci* for the development of ALC contributes to improving the risk prediction for cirrhosis and defining disease subtypes, representing an inspiring contribution to precision medicine.
